# Scat piling and strong frugivory of the Balearic lizard, *Podarcis lilfordi* (Günther, 1874)

**DOI:** 10.1186/s40850-022-00125-w

**Published:** 2022-05-09

**Authors:** Ana Pérez-Cembranos, Valentín Pérez-Mellado

**Affiliations:** Department of Animal Biology, University of Salamanca, Salamanca, Spain

**Keywords:** *Podarcis lilfordi*, Lacertidae, Foraging, Diet, Balearic Islands, Plant consumption

## Abstract

**Background:**

In lacertid lizards from Mediterranean islands, frugivory is common, particularly under prey scarcity, a characteristic of small islands. In several populations, the diet of the Balearic lizard, *Podarcis lilfordi,* is extremely variable and includes fleshy fruits. However, frugivory is sporadic and there are very few examples of dominant fruit consumption.

**Results:**

We describe the existence of an extraordinary fruit consumption of a single plant species, the juniper, *Juniperus phoenicea*, by the Balearic lizard, *P. lilfordi*. In addition, for the first time in Lacertidae, we describe the existence of scat piling in the population of these lizards inhabiting Cabrera Island (Balearic Islands, Spain). Scat piling was detected in an isolated location with hundreds of scats deposited by several individuals at a particular place.

**Conclusions:**

The high population density of lizards at the island of Cabrera and the great versatility of foraging behavior of this species allows us to hypothesize that scat piles could act as an attractor for numerous individuals, that is, as inadvertent social information. If that hypothesis is correct, it would result in the concentration of several individuals foraging on a single or a few ripening plants. We cannot, however, rule out that individuals concentrated due to the scarcity of ripening plants in other areas, without any influence of the presence of several lizards, as attractors to the site. Our findings modify previous descriptions of the diet of the Balearic lizard in Cabrera made with smaller samples. In some places and periods of the year, frugivory on a single plant species can be extremely intense and only large sample sizes of scats allow to find these particular trends in the foraging ecology of insular lizards.

**Supplementary Information:**

The online version contains supplementary material available at 10.1186/s40850-022-00125-w.

## Introduction

Insectivory is the plesiomorphic condition of modern lizard species [1], even if the trend towards herbivory has been frequently observed during the evolution of Squamata [2]. However, the consumption of plant matter and, particularly, the consumption of fleshy fruits is frequent in insular lizards [3, 4]. It has been pointed out that frugivory can be a consequence of a low arthropod availability, as some studies suggest that islands can support fewer insect taxa, which might be less abundant than in equivalent continental areas [5–9].

Lizard diets are normally analysed based on low sample sizes from each population, and samples are often obtained in a particular season or year [3 and references therein]. Thus, dietary studies normally represent a snapshot of the true diet of a given species, considering that diet composition can differ among seasons and years [10–12]. A previous study from a single locality, Aire Island (Menorca, Spain), inhabited by the Balearic lizard, *Podarcis lilfordi* (Günther, 1874), showed that there is a very large variability among years, areas, and seasons, precluding a reliable description of the diet with small sample sizes [13].

**Fig. 1:**
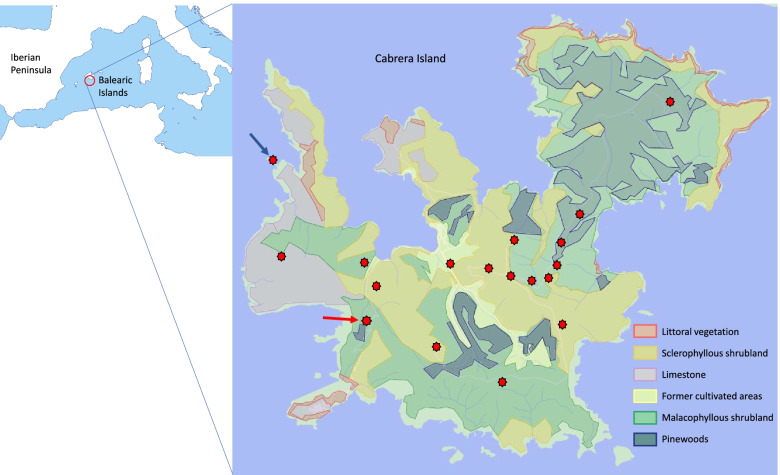
Situation of Cabrera Island (left), main vegetation types (simplified from Rita & Bibiloni, 1993) and sampling sites (red points) of lizard scats. The red arrow indicates the location of the scat pile within a malacophyllous shrubland of Western part of the island. The blue arrow indicates the situation of the sampling point corresponding to littoral vegetation type (Morro den Tià peninsula). Pinewoods were mixed with malacophyllous shrublands and were not separately sampled

Cabrera Island, the main island of the National Park of Cabrera, in the southern coast of Mallorca (Balearic Islands, Spain, Fig. 1), exhibits one of the largest populations of *P. lilfordi* [14, 15]. Pérez-Cembranos et al. [15] described the diet of the Balearic lizard from a sample of 318 faeces obtained in four different years: 2004, 2016, 2017 and 2018 and from four different months: June, July, August and October. It was showed that there is a diffuse variability in the diet at different areas of Cabrera Island, but without significant differences among these areas, and even without significant differences among coastal islets and Cabrera Island [15]. In addition, a significant negative correlation was found between prey diversity and the amount of plant consumption among populations. Finally, the authors did not find any relationship between plant consumption and plant species diversity available in each population [15].

**Fig. 2:**
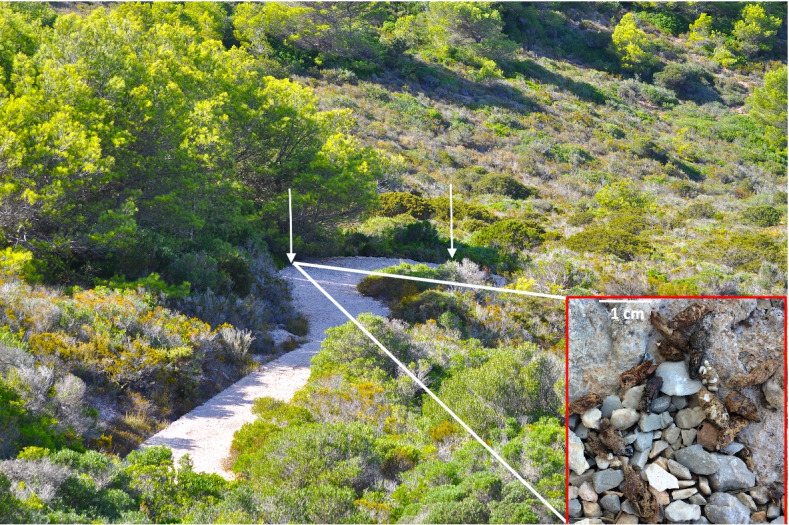
The scat pile found in Cabrera Island. White arrows indicate the limits of the narrow scat pile (see dimensions in the text). Behind the left turn of the track, we can see a slope densely covered by malacophyllous shrubs, including *Juniperus phoenicea*. On the lower right corner, a detail of the accumulation of faeces, with 16 scats in a very reduced area

During August 2020 we sampled Cabrera Island, covering the whole surface and major vegetation types of the island (Fig. 1) and collected a large sample of faeces from different localities. Surprisingly, in one location, we found a scat pile (Fig. 2), that is, a very large accumulation of scats in a single small place [16]. This accumulation of faeces in piles is a common behavior of several mammal species and was also described in some lizards [17]. For example, the production of piles of scats was described in banded geckos that employ preferred sites for defecation, speculating about the role of these scat piles as signposts [18]. The genus *Egernia* is also well-known as a producer of scat piles close to the refuge sites [17, 19, 20]. It was suggested that scat piles can facilitate avoidance of predator detection [21], but the main role is probably chemical communication [17]. In lizards, scat piles can play a role in recognition of territory boundaries or in social cohesion within a group of lizards [17, 19, 22]. Lizard frugivory is particularly common in islands. Valido and Olesen [4], reported 470 species from 27 different families that include fleshy fruits in their diet. 62.4% of these species inhabit islands. Thus, lizard frugivory can be considered an “insular phenomenon” [4]. The objective of this study was to describe the diet of the Balearic lizard during August 2020 in Cabrera Island. During this study, the existence of scat piling behaviour was found. At the same time, we try to analyse the potential bias of diet description because of the use of small sample sizes and a limited geographical coverage present in previous studies [15].

## Results

From 610 faeces, we identified 1237 food items. The overall August diet of *P. lilfordi* from Cabrera Island includes 17 food types (Table 1). Only ants and terrestrial Isopoda are relatively common prey (Table 1). In two scats from a locality of malacophyllous shrublands, we found remains from juveniles of *P. lilfordi*. The sporadic consumption of young lizards is common in the diet of the Balearic lizard [3, 13, 23]. There is an astounding dominance of seeds from juniper, *Juniperus phoenicea* (Cupressaceae) fruits. From 737 seeds (Table 1), only one single seed was identified as *Juniperus oxycedrus* (Cupressaceae), from the sample scats of sclerophyllous shrublands; 16 seeds from the scat pile were from *Pistacia lentiscus* (Anacardiaceae). In addition, 4 seeds from *Phillyrea media* (Oleaceae) and 19 seeds from *Rubia peregrina* (Rubiaceae) were detected in faeces from the sample of former cultivated areas (Table 1). The remaining seeds belonged to *J. phoenicea.* In fact, seeds make up almost 60% of the diet of *P. lilfordi* on Cabrera Island and are present in more than 86% of the scats. This dominance is common to samples from all vegetation areas, except for the sample from littoral vegetation (see below). In the two samples of the scat pile, almost all scats contained juniper seeds (Table 1). Because lacertid lizards do not chew their food items, seeds remain intact. These results suggest that the Balearic lizard is probably a legitimate seed-disperser of these plant species in Cabrera Island.

**Table 1:** Summer diet (2020) of the Balearic lizard in Cabrera Island

Taxa		Overall diet				Scat pile A				Scat pile B			Sclerophyllous shrublands				Malacophyllous shrublands				Limestone vegetation				Littoral vegetation				Former cultivated areas			
	frequency		presence		frequency		presence		frequency		presence		frequency		presence		frequency		presence		frequency		presence		frequency		presence		frequency		presence	
	n	n%	np	%p	n	n%	np	%p	n	n%	np	%p	n	n%	np	%p	n	n%	np	%p	n	n%	np	%p	n	n%	np	%p	n	n%	np	%p
Gastropoda	2	0.16	2	0.33	1	0.22	1	0.38									1	0.35	1	0.88												
Pseudoscorpionida	1	0.08	1	0.16													1	0.35	1	0.88												
Araneae	8	0.65	8	1.31	1	0.22	1	0.38					2	0.82	2	1.89	4	1.4	4	3.54									1	1.56	1	4.55
Isopoda	131	10.59	130	21.28	39	8.63	38	14.39	14	12.61	14	22.58	23	9.47	23	21.7	25	8.77	25	22.12	5	17.24	5	31.25	20	37.74	20	74.07	5	7.81	5	22.73
Diplopoda	2	0.16	2	0.33	1	0.22	1	0.38																	1	1.89	1	3.7				
Dictyoptera	6	0.49	6	0.98	4	0.88	4	1.52	1	0.9	1	1.61					1	0.35	1	0.88												
Isoptera	2	0.16	2	0.33					1	0.9	1	1.61	1	0.41	1	0.94																
Hemiptera	40	3.23	35	5.73	5	1.11	5	1.89	1	0.9	1	1.61	12	4.94	10	9.43	18	6.32	15	13.27	1	3.45	1	6.25	3	5.66	3	11.11				
Diptera	20	1.62	20	3.27	4	0.88	4	1.52	2	1.8	2	3.23	5	2.06	5	4.72	9	3.16	9	7.96												
Lepidoptera	4	0.32	4	0.65													4	1.4	4	3.54												
Coleoptera	31	2.51	29	4.75	4	0.88	4	1.52	1	0.9	1	1.61	1	0.41	1	0.94	10	3.51	10	8.85					14	26.42	12	44.44	1	1.56	1	4.55
Hymenoptera	11	0.89	8	1.31					1	0.9	1	1.61	2	0.82	2	1.89	6	2.11	4	3.54					2	3.77	1	3.7				
Formicidae	236	19.08	137	22.42	34	7.52	26	9.85	9	8.11	7	11.29	72	29.63	44	41.51	81	28.42	38	33.63	10	34.48	6	37.5	12	22.64	8	29.63	18	28.13	8	36.36
Arthropoda undet.	3	0.24	2	0.33	1	0.22	1	0.38									2	0.7	1	0.88												
Larvae	1	0.08	1	0.16	1	0.22	1	0.38																								
Seeds	737	59.58	529	86.58	357	78.98	260	98.48	81	72.97	62	100	125	51.44	91	85.85	121	42.46	85	75.22	13	44.83	11	68.75	1	1.89	1	3.7	39	60.94	19	86.36
Podarcis	2	0.16	2	0.33													2	0.7	2	1.77												
Total	1237	100	610		452	100	264		111	100	62		243	100	106		285	100	113		29	100	16		53	100	27		64	100	22	

Frequency, n: prey frequency of each taxon, n%: percentage of prey of each taxon in relation to prey number. Presence, np: number of scats in which each taxon is present and %p: percentage of scats in which each taxon is present. The number of prey items from a given taxon is estimated from the count of significant elements. Scat piles A and B corresponding to the two samples obtained in the western latrine within malacophyllous vegetation (see more details in the text)

The scat pile showed the highest percentage of seeds from *J. phoenicea* in comparison with scats from the remaining vegetation areas*.* This scat pile was detected on a narrow track that leads from Cabrera Bay to the Ansiola Peninsula (Fig. 1). The area (Fig. 2) was a narrow edge of a track raised around 50 cm above the ground and whose outer edge is formed by a stone wall with numerous rocky crevices, excellent refuges for lizards. The surface covered with faeces was a narrow rectangle of 7 m long and 0.2 m wide, that is, an area of 1.4 m^2^ (Fig. 2). The clear and gravel-covered path, and the rocks on its outer edge, are optimal sites for thermoregulation. The area is partially shaded by *Pinus halepensis*, with some shrubs of *J. phoenicea* that, during August 2020, were fully ripening. On a first visit (24th August), we obtained a very large sample of 264 faeces (scat pile A). Only 6 days later, we were able to find 62 additional faeces at the same place. Moreover, on 29th August, there was a strong storm with heavy rain on Cabrera. Heavy rain washes the ground, dragging previously deposited faeces, especially when the ground has some slope (pers. obs.), such as the area where the scat pile was found (Fig. 2). Thus, this second sampling of 62 faeces was deposited during a very short period of time, around 2 days and, consequently, by several individual lizards. In addition, that speed of deposition suggest a fast accumulation scats from the first sample.

The comparison among the six vegetation zones of the island and the two samples of the scat pile, showed that diet composition was significantly different among samples (permutational MANOVA, F_6,606_ = 23.27, *p* = 0.0009). Pairwise comparisons indicate a similar diet composition between the two samples of the scat pile (pairwise adonis, F = 0.66, *p* = 0.575). In the remaining paired comparisons, the scat pile was significantly different to the rest of samples, including the sample from malacophyllous vegetation (*p* < 0.005, Table 2), the vegetation area where the scat pile was found. Among vegetation areas, we only found significant differences between the diet of the area of littoral vegetation and the rest of vegetation areas (Table 2).

**Table 2:** Vegetal matter consumption by the Balearic lizard from different vegetation areas

	Scat pile A	Scat pile B	Malacophyllous	Sclerophyllous	Limestone	Cultivated
Scat pile A 94.15 ± 1.08 (264)						
Scat pile B 97.61 ± 0.93 (62)	Z = 5.38 *p* = 0.589					
Malacophyllous 67.84 ± 4.13 (102)	Z = 6.637 ***p*** **= 3.18 × 10**^**−11**^	Z = 4.333 ***p*** **= 1.47 × 10**^**−5**^				
Sclerophyllous 79.85 ± 3.29 (117)	Z = −5.052 ***p*** **= 4.35 × 10**^**−7**^	Z = −3.088 ***p*** **= 0.002**	Z = 1.569 *p* = 0.116			
Limestone 70.56 ± 11.37 (16)	Z = 1.605 *p* = 0.108	Z = −1.202 *p* = 0.229	Z = 1.34 *p* = 0.18	Z = -0.554 *p* = 0.579		
Cultivated 83.14 ± 7.36 (22)	Z = −1.381 *p* = 0.167	Z = −0.929 *p* = 0.352	Z = 1.987 *p* = 0.046	Z = 1.095 *p* = 0.273	Z = 0.324 *p* = 0.745	
Littoral 7.96 ± 3.46 (27)	Z = 9.905 ***p*** **= 3.95 × 10**^**−23**^	Z = 8.349 ***p*** **= 6.83 × 10**^**− 17**^	Z = − 5.671 ***p*** **= 1.41 × 10**^**−8**^	Z = 6.745 ***p*** **= 1.52 × 10**^**− 11**^	Z = 5.033 ***p*** **= 4.81 × 10**^**− 7**^	Z = 5.90 ***p*** **= 3.62 × 10**^**−9**^

In the first column we give the average percentage volume per scat (mean ± SE) and, within parentheses, the number of scats analyzed. For each pairwise comparison, we give the Z-value and *p*-value of the post hoc Dunn test (see more details in the text). In bold, significant *p*-values

The consumption of vegetal matter was significantly different among samples (Kruskal-Wallis test, 𝜒^2^ = 134.25, d.f. = 6, *p* = 2.2 × 10^− 16^), with the lowest consumption of plant matter in the littoral vegetation area and the highest consumption in both samples of the scat pile (Table 2). Except in the case of littoral vegetation, juniper seeds make up this large proportion of vegetal matter in scats. The sample from littoral vegetation was obtained at Morro den Tià (Fig. 1), an almost completely isolated peninsula from western coast of Cabrera, where junipers are absent and where the diet and other ecological traits of lizards are like those of coastal islets around Cabrera [15].

## Discussion

The omnivorous diet of the Balearic lizard is well-known [3, 13, 15, 23]. In all studied populations, we detected an important plant consumption. An example is the case of frugivory on *Helicodiceros muscivorus* (Araceae) in Aire Island (Menorca, Balearic Islands), where *P. lilfordi* is the main seed-disperser [24]. However, to our knowledge, this is the first case where a single plant species, *J. phoenicea*, is so intensively consumed during the summer season.

We detected only few differences in the diet of the Balearic lizard through the different vegetation areas of Cabrera Island, except for our more surprising finding, the spectacular consumption of juniper fruits. The Balearic lizard is likely an important seed-disperser of this plant in Cabrera Island. We must note that a dramatic change of vegetation cover of Cabrera Island took place during the last century, with an increase in the area covered by trees and shrubs, after the eradication of large domestic herbivores such as goats [25]. Our results provide further evidence for the trophic generalism in foraging behaviour of *P. lilfordi*.

The cones of *Juniperus* species, functionally analogous to angiosperm fleshy fruits [26], are intensively consumed by lizards. *J. phoenicea* is mainly considered a bird-dispersed species [27, 28] and Traveset [29] mentions that it is an endozoochorous plant species. Salvador [30] and Sáez & Traveset [31] showed that the Balearic lizard eats the fruits of *J. phoenicea*. Additionally, Pérez-Cembranos et al. [15] showed that seeds of *J. phoenicea* are currently present in scats from *P. lilfordi* of Cabrera. It is also known that the sister species of the Balearic lizard, the Ibiza Wall lizard, *Podarcis pityusensis*, is the main seed disperser of *J. phoenicea* in some populations [32]. Thus, this food item is not uncommon in the diet of lizards from the Balearic Islands. The uncommon fact is the strong intensity of consumption observed during August 2020 in Cabrera Island. In this way, the inclusion of two samples from a single locality of Cabrera Island, the scat pile, led to a dramatic change in the overall summer diet of the Balearic lizard, with a dominance of one plant species and a single prey item, terrestrial Isopoda.

In all populations of the Balearic lizards surveyed during last 30 years, we observed a random distribution of scats only slightly concentrated in small rock crevices, but never in a number higher than four or five faeces [13, 23]. Surprisingly, our observations from 2020 in Cabrera Island showed an extraordinary concentration of scats in a particular location, the edge of a track to a lighthouse in the Western part of the island (Fig. 2). Thus, our data show that free-living Balearic lizards form scat piles in some locations, at least in Cabrera Island. This extraordinary accumulation of scats in a single site consisted almost exclusively of seeds of a single plant species. This is probably the consequence of an intense foraging of several individual lizards on a few nearby plants with ripening fruits.

As we argued above, scat-piling was the result of the foraging activity of several individual lizards. Social foraging is not uncommon on abundant food items, where multiple lizards feed simultaneously [33, 34]. In the Balearic lizards we have several observations of aggregations of lizards around food resources, as it was observed in Aire Island [13]. Therefore, it is not difficult to imagine the concentration of numerous lizards in one or more junipers with ripe fruits and the intense collective exploitation of this food resource during several consecutive days. What is no longer so evident is the reason why many individuals deposited their faeces on a small area, leading to the formation of a scat pile. The place occupied by the scat pile can be particularly favorable as a place for thermoregulation [35] and/or, as a rocky refuge, close to a temporarily abundant food resource. In well-studied lizard species of the genus *Egernia*, scat-piling is a common behavior, with the accumulation of scats close to basking sites [17, 20]. In some species, as *Egernia cunninghami*, an accumulation of 40–50 scats was recorder [17]. But we do not know any species where scat piles can accumulate hundreds of faeces in a single spot, as we recorded in Cabrera Island.

Communal faeces deposits can have a social role, at least in some lizard species. However, this role was discarded in the case of some gekkonid lizards as *Nephrurus milii* [36], where scat piling is not apparently related with sociality. In fact, even employing a broad definition of sociality, no species of lacertid lizards have been found to form stable aggregations [37]. Moreover, despite of the observations that faeces of some species, such as *Iberolacerta cyreni*, are deposited on visually conspicuous sites and have an aggregated spatial distribution [38], there is no previous information on the existence of scat piling in lacertid lizards.

The area of faecal accumulation observed in Cabrera Island is not a particularly visually detectable place. This scat pile was not located at the top of a rocky site, but on the edge of a gravel path, where faeces accumulated in depressions of the ground and are not particularly conspicuous (Fig. 2). What was especially striking was the accumulation of faeces and not their situation on the ground (Fig. 2). From that point of view, we do not have any conclusive evidence that the function of scat-piling in this population of the Balearic lizard is to increase its visual detectability for conspecifics, as has been shown in other lacertid lizards [39].

The accumulation of faeces in a scat pile can by itself be an attractive factor for other lizards. A small area around the scat pile could have a high availability of ripening cones of *J. phoenicea*. It is a current fact that in Mediterranean habitats there are important differences in cone availability among nearby areas [26]. The strong versatility of foraging behaviour of the Balearic lizard [13], allows this species to find locations with a particular abundance of a trophic resource, following behavioural “resource tracking”, as showed in frugivorous birds [26]. The faeces, mainly composed from seeds and remains of *J. phoenicea* ripen cones, have a penetrating odor due to highly volatile terpenoids originating from berries [40], detectable at a great distance. It is well-known that lizards can use social information to learn how to enhance their foraging opportunities [41–44]. Even with high lizard densities, conspecifics can act as informers, rather than competitors, sending out Inadvertent Social Information (ISI), sensu [45]. In this way, chemical signals from the scat pile could act as inadvertent social information, attracting conspecifics to the area. Attracted conspecifics could detect shrubs with ripe fruits that are close by with a greater probability. This attraction, thanks to inadvertent chemical signals, would lead to a positive feedback loop: lizards exploit plants with ripening fruits for several days or weeks while using rocky crevices in the area immediately adjacent to the scat pile as safe refuges [46]. By doing so they continuously accumulate faeces and increase the attractiveness of the place for new lizards. Importantly, similar to some frugivorous birds, the strong reliance of the Balearic lizard on junipers we recorded in summer of 2020 in Cabrera Island, could be an outlier of that year and population. Thus, we can expect extremely variable outcomes in time and space [24], with strong differences among years or even sites of Cabrera Island.

From the results obtained in this study, it is tempting to deduce that fleshy fruits of certain shrub plants, such as *J. phoenicea*, are always an essential element of the summer diet of the Balearic lizard in Cabrera Island. However, the spectacular contribution to the diet of this plant species has only become apparent in the summer of 2020. In previous studies, the seeds of *J. phoenicea* have never been an important element of the diet [15, 29, 30]. Even if there is a wide distribution and abundance of *J. phoenicea* in Cabrera Island [25], fruit resources can be extremely patchy and each year ripening shrubs can be concentrated in some locations.

In many fruit-consuming vertebrates that are primarily insectivorous, the importance of fruits in the diet undergoes annual variations. In fact, it is probable that in *P. lilfordi*, the intensity of consumption of the fruits of a given species, such as *J. phoenicea*, is a local and/or seasonal phenomenon, without a direct relationship with the abundance or the availability of these fruits. In their review, Valido & Olesen [4], pointed out that their results are only the tip of the “lizard-plant seed dispersal iceberg”. We agree with this view, but at the same time, we think that frugivory can be extremely variable throughout years and locations.

## Conclusions

The Balearic lizard, at least in Cabrera Island, showed a very intense frugivory at the end of summer, focusing its foraging behavior in a few ripening plants. This behaviour is not common in lacertid lizards and probably it is only present in some populations and years. As a result of strong frugivory by several lizards concentrated on a reduced area, a scat pile was formed in a particular site. This is the first case of scat piling known in Lacertidae. We propose that this scat pile can be employed by lizards as Inadvertent Social Information (ISI), allowing a positive feedback in increasing concentration of lizards around ripening plants.

## Methods

### Study area and species

Cabrera gran is the main island of Cabrera Archipelago, in Southeastern Mallorca (Balearic Islands, Spain). Cabrera gran has a surface of 1154.75 ha, with a maximum altitude of 173 m.a.s.l. [47]. The island is covered by a Mediterranean vegetation with a wide variety of vascular plants [48]. Cabrera has been inhabited for centuries and has suffered strong human pressure, with the exploitation of various crops and with the pressure derived from the introduction of domestic livestock such as goats and sheep, the manufacture of charcoal and other uses [48, 49]. Fortunately, today, as a part of a National Park, the island is strictly protected.

The Balearic lizard, *Podarcis lilfordi* (Squamata, Lacertidae) is a medium-sized Endangered lizard that inhabits the coastal islets of Menorca, Mallorca, and Cabrera Archipelago [50]. This lizard reaches high densities in Cabrera Island [14, 15]. Balearic lizards are active foragers that hunt insects and other invertebrates, but they also consume vegetal matter, carrion, conspecifics, or leftovers carried by visitors [3].

### Dietary study

From the 23rd to the 31st of August 2020, a total of 610 scats were obtained directly from the ground. Because trophic availability could be different at different areas of a large island [13], we sampled Cabrera Island considering the main vegetation types (Fig. 1, [51]). We made several transect along vegetation types, employing allowed narrow tracks. We tried to employ a similar time searching at each vegetation area, even if some areas hold lower lizard densities and searching for faeces can be a longer task. According to these vegetation types, scats from 18 sampling sites were grouped into samples from sclerophyllous shrublands, malacophyllous (i.e., plants living in dry regions and having soft or fleshy leaves in which water is stored) shrublands, limestone vegetation, littoral vegetation, and former cultivated areas (Table 1). In one locality of malacophyllous vegetation we found a scat pile with an extraordinary accumulation of scats (Fig. 2). Two samples from this scat pile were obtained, on 24th August 2020 (scat pile A sample, Table 1) and on 31st August 2020 (scat pile B sample, Table 1, Fig. 2). These vegetation areas are relatively recent and caused by large changes in the vegetational composition during the last two centuries [25].

Faeces were analyzed under a binocular dissecting microscope. In lizards, diet reconstruction based on a meticulous scat analysis has been found to be highly comparable to those diet reconstructions based on gastric contents removed from dissected stomachs, with soft-bodied prey and particularly insect larvae, being equally represented in faeces and gut contents [52]. Each scat was spread in a thin layer of less than 0.5 mm over the entire surface of a Petri dish, with some drops of 70° ethylic alcohol. The percentage of vegetal matter was visually estimated according to the surface occupied by vegetal remains in the Petri dish. Plant families were arranged according to [53, 54]. Prey remains were identified up to Order or, on occasion to Family level. Prey number for each scat was conservatively estimated by counting only easily identifiable remains (see [13] for more details).

The diet of lizards living at different vegetation areas was compared with a permutational multivariate analysis of variance (permutational MANOVA, [55]), using the ‘adonis’ function from ‘vegan’ R package [56]. The multivariate homogeneity of group dispersions (variances) was tested with the function ‘betadisper’ from ‘vegan’ package, a multivariate analogue of Levene’s test for homogeneity of variances. We estimated and compared diet diversities using the approach proposed by Pallmann et al. [57]. Instead of describing diet diversity through a given index as, for example, Simpson or Shannon indices, we converted these “raw” indices into “true” diversities which all belong to one and the same mathematical family. That is, regarding different measures as special cases of Hill’s general definition of diversity measures [58]. In this way, to study differences in diversity among diets of different sites, we performed two-tailed tests for integral Hill numbers. This selection included the transformed versions of the three following indices: the species richness index, H_sr_ (q = 0), the Shannon entropy index, H_sh_ (q → 1) and the Simpson concentration index, H_si_ (q = 2, [59]). All comparisons among diversities were made with Tukey-like contrasts employing a resampling procedure. We did 5000 bootstrap replications to obtain reliable *p*-values [60]. Methods described here are implemented in R package “simboot” [61] and are fully described in [58]. Due to the heterogeneity of variances among samples (Fligner test, 𝜒^2^ = 307.82, d.f. = 6, *p* = 2.2 × 10^− 16^), the percentages of plant matter in the diet were compared with a Kruskal-Wallis test and pairwise comparisons were done with Dunn tests from R-package “FSA” [62]. All calculations were done in R version 4.0.3 [63].

## Supplementary Information

**Additional file 1.** Raw data. File with all the data used and analyzed in this manuscript.

## Data Availability

All data generated or analysed during this study are included in this published article (Additional file 1).

## Abbreviations

ISI: Inadvertent Social Information
m.a.s.l.: Meters above sea level
